# Evaluation of high school-based dengue solution model in Southern Thailand: a community participatory action research

**DOI:** 10.1186/s12889-024-20767-4

**Published:** 2024-11-28

**Authors:** Charuai Suwanbamrung, Sandeep Kumar Mehraj, Melkamu Worku Kercho, Muhammad Haroon Stanikzai, Temesgen Anjulo Ageru, Jiraporn Jaroenpool, Panatda Pibul, Shamarina Shohaimi, Eskinder Israel

**Affiliations:** 1 Public Health Research, School of Public Health, Walailak University, Nakhon Si Thammarat, 80160 Thailand; 2 School of Allied Health Sciences, Walailak University, Nakhon Si Thammarat, 80160 Thailand; 3 School of Public Health, Walailak University, Nakhon Si Thammarat, 80160 Thailand; 4 Department of Biology, Faculty of Science, University Putra Malaysia, Selangor, 43400 Malaysia; 5 Excellent Center for Dengue and Community Public Health: EC for DACH, Walailak University, Nakhon Si Thammarat, Thailand; 6 Department of Public Health, Faculty of Medicine, Kandahar University, Kandahar, Afghanistan; 7 School of Public Health, College of Health Science and Medicine, Wolaita Sodo University, Wolaita Sodo, 130 Ethiopia

**Keywords:** High school-based program, Participatory, Dengue, Dengue solution, Dengue prevention and control

## Abstract

**Introduction:**

By the time the globe started to tackle the COVID-19 pandemic, Southeast Asian countries had faced an increased dengue incidence, which has eventually become an important public health problem. However, effective and sustainable disease control measures in the area are still lacking. Therefore, the current study is aimed to evaluate the development and implementation of high school-based dengue solution model in Southern Thailand.

**Methods:**

Integrated community participatory action research (CPAR) was employed using preparation, planning, implementation, and evaluation. Data was collected using quantitative and qualitative methods from high school students. Descriptive statistics such as frequency and percentage, chi-square and fisher’s exact test were used to summarize and compare quantitative data before and after intervention. Similarly, qualitative data was collected through interviews and focus group discussion (FGD) and then analyzed through thematic analysis.

**Results:**

Two hundred and thirty-nine (96.3%, *n* = 239/248) and 232 (93.5, *n* = 232/248) participants were included in the interventions before and after, respectively. School-based dengue prevention was developed with input from a variety of stakeholders, including students, community leaders, health educators, district officials, and community health volunteers. As demonstrated by pre- to post-test results, students understanding of dengue and the larval indices surveillance system has increased. Students who received the training were not only inspired but created a sense of community responsibility with a high commitment to teaching and sharing information in their circle to enhance overall community wellbeing. Being female and higher educational attainment was associated with students understanding of dengue and larval indices surveillance.

**Conclusion:**

This participatory action research not only improved students' understanding of dengue but also empowered them to be proactive in various community health initiatives. The positive correlation between educational attainment and students understanding of dengue solution and larval indices surveillance underscores the need for tailored educational interventions that address diverse learning needs within the community. Collaborative efforts to establish dengue health information center based at primary schools and above can better improve reduction of dengue incidence.

**Supplementary Information:**

The online version contains supplementary material available at 10.1186/s12889-024-20767-4.

## Introduction

Globally, more than 400 million dengue cases were reported each year, most of them were from urban and semi-urban regions of tropical and subtropical zones [1]. By the time the globe started to tackle the COVID-19 pandemic, Southeast Asian countries had faced an increased dengue incidence, which has eventually become an important public health problem. The Asia continents, particularly, East Asia countries reported the highest number of COVID cases, which increased by 46% from 2015 −2019 [2]. The sudden emergence of COVID-19 along with the rise of dengue cases in the COVID era remained a double burden and had a significant impact on all aspects of the community wellbeing [3, 4]. The new COVID-19 pandemic measures were effective in preventing dengue transmission between cities and countries (since the mobility of individuals was temporarily limited) and greatly reduced co-circulation between various serotypes or genotypes. Growing evidence shows once after the dengue epidemic occurred, their distribution remains relatively stable and become resistant to COVID-19 methods [5]. This emphasizes the need for an accurate forecasting technique. Public health measures of COVID-19 such as restricting movement, closing schools and offices was surprisingly resulted in a significant drop of dengue cases in the cases of Sri Lanka [5]. However, this did not found to work for Peru and other dengue endemic countries as evidenced by the million rose of dengue cases during the pandemic despite all the COVID-19 prevention measures [5]. Conversely, no rise or decline in dengue cases over time was reported from Malaysia and Singapore with the implementation of COVID-19 measures [6].

Various prior studies targeted at school-based dengue prevention (aimed at closing the schools and limiting mobility during COVID-19) indicated having basic knowledge toward dengue among students has significantly reduced the dengue cases and decreased the risk of dengue among students as well as promoted behavioral change [7, 8]. This goes in line with the evidence from Sri Lanka and Thailand that demonstrated school-based dengue intervention decreased dengue incidence [9, 10]. Several lessons were learned from COVID-19 lockdown to enhance dengue control mechanisms at home and community levels. In the areas of high dengue incidence, maintaining effective mosquito control tactics were recommendable and worthy though, the effect of COVID-19 preventive measures on mosquito-borne illnesses remains somewhat uncertain [11].

Nabon, one of the districts in the southern part of Thailand and has been identified as a dengue risk area with reported dengue mortality rate of 201, 218, 267, 350, and 533 per 100,000 populations in the past five years (2015–2019) respectively before the COVID-19 pandemic. These reported rates exceeded threshold set by Thai Ministry of Public Health (TMOPH) (50 cases per 100,000 population) and considered as high-risk area for dengue outbreaks, particularly for the children under 15 years of age according to a provincial health office report [12]. One prior study done in Kaewsan district showed nearly one-third of the students had poor knowledge toward about dengue prevention, and the schools also lacked clear activities and networking connections [13]. Similarly, findings from Muslim community of Saturday and Sunday religious schools students showed students' basic knowledge toward dengue was significantly higher after the provision of an intervention than before [14]. This underscores the need to implement a full range of vector control interventions to decrease the risk of vector-borne diseases circulating in the school and community. To reduce this risk, Kaewsan Sub-district Administration Organization (KSAO) and Excellence Center for Dengue and Community Health (ECDCH) has been working in four schools-based project to develop a model that can enhance the system and reduce further dengue incidence using a school-based prevention approach. This project aims to assess students’ understanding of the dengue solution (UDS) and understanding of larval indices surveillance system (ULISS). UDS is defined as the capacity of students to comprehend dengue prevention, control, and self-care practice and ULISS refers to the capacity of students to comprehend the larval indices that characterize the larval indices surveillance system processes and the larval index levels.

While there were good surveillance measures in place in village and primary care unit (PCU) in Thailand, such as ongoing prevention campaigns, household (HH) dengue surveys, and destruction of mosquito breeding sites, little focus was given to the school environment and high school students [15]. Developing effective prevention models that involved key stakeholders was found to increase students’ knowledge toward dengue decreases their risk [16]. As to the knowledge of researchers, limited studies were focused on UDS and ULISS among high school students. Furthermore, effective and sustainable disease control measures in the area are still lacking. Therefore, the current study is aimed at evaluating development and implementation of high school-based dengue solution models in Southern Thailand.

## Materials and methods

### Study design and study setting

Integrated community participatory action research (CPAR) was employed among high school students in Nabon district, Nakhon Si Thammarat province, Southern Thailand from March to May 2022. We involved key stakeholders from village, two primary care units (PCU), sub-district administrator, and village health volunteers (VHV). Students (grades 10, 11 and 12) and their leaders were also participated in the intervention. The key stakeholders were selected purposely based on the inclusion criteria of the study. After informing them of the aim of the study and getting informed consent (written or oral), they were enrolled into the study.

### Development step

 We employed four basic steps to develop a high school-based dengue solution model appropriate for the local context. These are preparation, planning, implementation and evaluation.

### Step −1 Preparation

Firstly, we prepared and formed a strong stakeholders’ committees meeting that involved several teams (one SAO administrator, one district health officer head, two health educators and 10 students’ leaders, four community health officer and 04 community leaders). The aim of the meeting was to brief the aim and activities of the model to the participants on how to solve the rising dengue problem and the role of each stakeholder in the intervention. Secondly, we created a student-leaders club (where students can lead themselves) and gave educational training through lectures, role plays and in the form of video. The components of training encompassed assessing understanding of dengue illness, understanding larval indices surveillance system, how to get help, and how to teach others. This club consists of 5 students per each club in the school named "Mosquito Control and Dengue Prevention Club" or “areas of three zones” where intervention takes place among students. It was primarily established to carry out every activity of the school to support the school surveillance system. The roles of each student’s activities were followed, promoted and encouraged in the intervention process.

### Step-2 Planning

Following preparation phase, careful planning was done with key stakeholders on the activities to be done within 12 months. In this phase, the problems were carefully assessed, determined and prioritized. The planned activities were to meet the school’s principal and other stakeholders, including health educators and community leaders every monday for 12 weeks. Efforts to promote dengue solutions in the school in front of flagpole and putting dengue information board were also planned. Pictures related to the dengue solution, local dengue slogan contest and dengue awareness campaigns through walking and drama plays were planned to gain wider attention.

Assessment of school environment for the solution plan and provide feedback was done to develop effective prevention model in the school. The tool included various parameters such as student, ecological, water container and larval index related characteristics.

Students’ UDS and ULISS levels were important variable in this study and assessed according to Bloom’s revised taxonomy for learning. “Understanding” in this taxonomy has six cognitive levels such as knowledge, comprehension, application, analysis, synthesis, and evaluation. To reduce dengue incidence, students surveyed the larval indices in the school once per week (every Monday) for the full of the intervention phase in case they need help, get communicated with school directors and health educators in the school.

Both UDS and ULISS training was prepared for students and for the stakeholders to have awareness and knowledge to predict as well as to teach their families and others in their circle. The training was mainly focused on comprehensive clinical manifestation of the dengue and larval indices and their level of understanding.

### Step 3-Implementation

Prior to the study, ethical approval was received. The main activities done in this phase were meeting with key stakeholders and school principal, provision of UDS and ULISS training to both students and stakeholders (including health educators) for 12 weeks (though in a separate due to difference in the level of understanding), assignment of responsibility (duties) to each stakeholder and students, following students and monitoring overall development of the model. This educational training was given every week for 03 consecutive months (a total of 12 weeks) and lasts 45–60 min. The research team explained and responded to the points raised in the discussion. The school health educator and student leaders assessed various aspects of the school’s environment and larval indices survey, such as drinking water containers, used water containers, the water containers in the bathroom and toilet, cupboard saucers in the cafeteria, vases, plant-related containers, and discarded containers around the school.

### Step −4 Evaluation

This phase was characterized by evaluating the feedback from the students and stakeholders who were involved in the model development before and after the interventions. It included brief meetings with involvement of key stakeholders such as researchers, public health professionals, health educators and all others who were involved in dengue prevention in the school. Steps of model development and its effectiveness before and after using intervention (model) were also evaluated. Additionally, stakeholders’ reflection along with students UDS and ULISS levels before and after were measured and compared to evaluate the effectiveness of newly developed interventions. Moreover, student's behavior to larval indices survey in their households and schools were evaluated.

### Questionnaires for assessment and evaluation

The questionnaires (10 UDS and 10 ULISS items) was adapted from the previous studies conducted at Lansaka district and modified based on the local context [17]. Three experts assessed the content validity index values, showing 0.82 for UDS and 0.88 for ULISS items. Similarly, internal consistency reliability values (Cronbach alpha) of UDS and ULISS of 30 students were 0.72 and 0.73, respectively. Based on Bloom’s cut-off score of 80%, we used mean scores for UDS and ULISS items to describe good and poor levels of students understanding. In this study, obtaining 80% or more correctly indicated a good understanding, whereas less than 80% indicated a poor level of students understanding. Similarly, unstructured questionnaires were prepared for leader students and stakeholders’ reflection on project’s relevance, such as their participation in the school activities including larval indices surveillance system (LISS), training program, and the model utility and inhibiting barriers, and the way forward.

To manage the number of students, sample size was calculated using G*Power software [18]. The parameters included were taken from chi-square test statistics such as:—an effect size of 0.25 with alpha (α) 0.05, and a confidence level of 95%. Similarly, we set the power (1-β) at 0.95, with a degree of freedom of 3 before and after the group (DF = (r-1) *(c-1) = (3–1) *(2–1) = 2) and obtained a final sample size of 248. Then, the final sample size obtained was divided into their respective grades based on proportional allocation (Grade 10 = 105, grade 11 = 76, and grade 12 = 67) from the total of 427 students (Grades 10 (*n* = 180), Grade 11 (*n* = 130), and Grade 12 (*n* = 117). The students were selected randomly without replacing them from the students list since there was a high volume of students. Additionally, informed consent from the participants was taken after providing detailed information about dengue and larval indices surveillance system.

## Data analysis

Descriptive statistical analysis was conducted to describe frequency and percentage. Additionally, chi-square and fisher’s exact test were used to compare UDS and ULISS level before and after the intervention. Larval index analysis (LIA) was analyzed using house index (HI), container index (CI), and breteau index (BI). CI refers to the percentage of water-holding containers infested with larvae and/or pupae while BI also tells you the number of positive containers per 100 houses inspected. Similarly, HI is defined as the percentage of houses infested with mosquito in given area per the total number of houses infested [19, 20]. However, Thai Ministry of public Health only suggests CI for the school larval indices system. The qualitative data from the focus group discussion (FGD) were analyzed through Braun and Clarke’s thematic analysis by thorough reading responses, coding keywords, assigning categories, interpreting the meaning of the quotes, and lastly determining the themes [21, 22].

## Results

### Overall development of high school-based dengue solution model

**Fig. 1:**
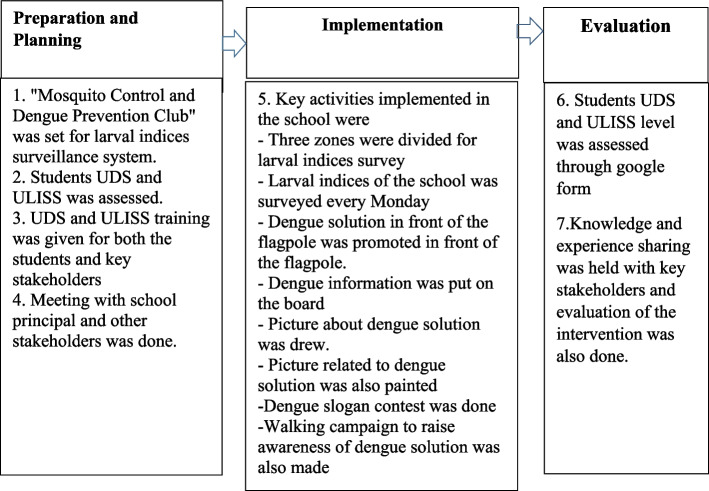
Development of high school-based dengue solution model on post-COVID-19 in Southern Thailand

The development process (preparation, planning, implementing and evaluating) of high school-based dengue solution model involved various stakeholders and included different activities (Fig. 1).

### Socio-demographic and dengue experience of students

Two hundred and thirty-nine (96.3%, *n* = 239/248, 96.3%) and 232 (93.5, *n* = 232/248) participants were included in the interventions before and after, respectively (Table 1). More than two-third (72%, *n* = 391/471) of the students were females and majorities (83.0%, *n* = 391/471) were in the age group of 15–17. It also showed unlike males, female students and those aged 18 years or below were higher in number across both measurement (pre and post) indicating good participation which is related to demographical condition of the country as well. In this study, majority of the participants received dengue training. The mean ± SD of the students age were 16.38 ± 1.02.

**Table 1:** A table showing students' sociodemographic characteristics before and after using the school-based model

Variables	Categories	n (%)			*χ2*
		**Before** (*n* = 239)	**After** (*n* = 232)	**Total**	
Sex	Male	71 (15.1)	61 (13.0)	132 (28.0)	0.41^*ns*^
	Female	168 (35.7)	171(36.3)	339 (72.0)	
Age	10–14	3 (0.6)	2 (0.4)	5 (1.1)	0.887^*ns*^
	15–17	197 (41.8)	194 (41.2)	391 (83.0)	
	≥ 18	39 (8.3)	36 (7.6)	75 (15.9)	
Frequency of home-based survey	Daily	11 (2.3)	12 (2.5)	23 (4.9)	0.18^*ns*^
	Weekly	48 (10.2)	65 (13.8)	113 (24.0)	
	Monthly	157 (33.3)	139 (29.5)	296 (62.8)	
	None	23 (4.9)	16 (3.4)	39 (8.3)	
Dengue training given	Yes	6 (1.3)	5 (1.1)	11 (2.3)	0.79^*ns*^
	No	233 (49.5)	227 (48.2)	460 (97.7)	
Students experience dengue illness in the past 12 months	Themselves	17 (4.7)	14 (3.9)	31 (8.6)	0.79^*ns*^
	Neighbor	43 (12.0)	43 (12.0)	86 (24.0)	
	Family member	9 (2.5)	11 (3.1)	20 (5.6)	
	Others	103 (28.7)	119 (33.1)	222 (61.8)	

*p* *p*-value of Chi-square test used unless otherwise noted. ^(a)^Fisher’s exact test was used. ^*^p < 0.05, *ns* Non-significant

### Understanding of dengue solutions (UDS) level among students

This intervention showed marked improvement in the students’ UDS level as seen from change in their responses from before to after the intervention in identifying signs and symptoms of dengue, treatment and all the prevention modalities (Table 2).

**Table 2:** Understanding of dengue solution among high school students before and after intervention

UDS Items	Correct understanding n (%)			OR	95%CI		P
	**Before** **n (%)**	**After** **n (%)**	**Total**		**Lower**	**Upper**	
1. A high-grade fever lasting up to 7 days, petechiae, and a painful enlargement of the liver are signs of dengue infection	187 (49.1)	194 (50.9)	381	1.42	0.89	2.25	0.139* ns*
2. Having a signs and symptoms of dengue indicate dengue viral infection	92 (36.1)	163 (63.9)	255	3.77	2.57	5.53	0.000***
3. A person living in a high-risk area and were infected with one dengue serotype can have lifelong immunity to that strain though vulnerable to other serotypes and could still be infected with other serotypes later too	103 (37.3)	173 (67.2)	276	3.87	2.61	5.72	0.000***
4. If a person isprotected from female *Aedes aegypti* bites, they will be safe from dengue	166 (47.3)	185 (52.7)	351	1.73	1.13	2.64	0.01**
5. Having a high fever lasting for 2–7 days, nausea, vomiting, and abdominal pain shows the patient is in the fever stage	127 (41.9)	176 (58.1)	303	2.77	1.87	4.10	0.000***
6. If a patients with dengue hemorrhagic fever presents with pain at right lower costal margin, they are showing signs of hepatomegaly	59 (26.6)	163 (73.4)	222	7.20	4.79	10.82	0.000***
7. If a patients with dengue hemorrhagic fever presents signs of shock from leakage of the plasma, they will have poor tissue perfusion, weak pulse, and narrowed pulse pressure	73 (31.2)	161 (68.8)	234	5.15	3.48	7.63	0.000***
8. If your neighbor presents with a signs of poor tissue perfusion, weak pulse, and clammy skin, you need to send them to the hospital	136 (64.5)	75 (35.5)	211	0.36	0.24	0.52	0.000***
9. Dengue patients should avoid taking aspirin or non-steroidal anti-inflammatory drugs because they may cause gastritis and subsequent massive gastrointestinal or hepatic injury	43 (21.3)	159 (78.7)	202	9.92	6.45	15.27	0.000***
10. If your neighbor presents with a high-grade fever on day 1, you give one paracetamol every 6 h and a tepid sponge bath	117 (41.2)	167 (58.8)	284	2.67	1.82	3.92	0.000***

*p* *p*-value of Chi-square test used unless otherwise noted. (a)Fisher’s exact test was used. ***p < 0.001 **p < 0.01 *p < 0.05 *ns* Non- significant, *OR* Odd ratio; * CI* Confidence interval

### Understanding of larval indices surveillance system (ULISS) among students

All the ULISS items among students increased after the intervention when compared with before the interventions. This shows significant increase in correct ULISS items understanding that will help them with dengue prevention. Their understanding was greatly improved in insecticide use and involvement of family leaders in the survey which is very important for dengue prevention both in their home and school as well (Table 3).

**Table 3:** Student's understanding of larval indices surveillance system (ULISS) among high school students

**Student’s ULISS items**	***Correct understanding n (%)***			**OR**	**95% CI**		***P***
	**Before** **(*n* =)**	**After** **(*n* =)**	***Total***		**Lower**	**Upper**	
1. The number of larvae of female *Aedes aegypti*s in the areas is determined by larval indices	65 (56.5)	50 (43.5)	115	0.73	0.48	1.12	0.155^*ns*^
2. Through container index (CI), the value for identifying a dengue outbreak iscalculated and then the number of water containers infested with larvae is surveyed	65 (61.9)	40 (38.1)	105	0.55	0.35	0.86	0.01^*^
3. Involvement of family leaders in the larval survey in household level every week is a key	63 (27.5)	166 (72.5)	229	7.02	4.68	10.53	0.00^***^
4. Insecticide Temephos and granulates may be used to eliminate larvae but not to eliminate mosquito eggs	17 (10.6)	143 (89.4)	160	20.98	11.98	36.72	0.00^***^
5. Use of lotion in the neighborhood works for the prevention of mosquito bites	74 (33.2)	149 (66.8)	223	4.00	2.72	5.87	0.00^***^
6. Eliminating mosquito breeding sites and endeavouring to diminish the scores for the larval indices around the house works for the prevention of massive occurrence of dengue among people, especially in the village	25 (65.8)	13 (34.2)	38	0.50	0.25	1.01	0.05^*^
7. Cleansing and scrubbing the edge of the container over the area that had water is effective in eliminating mosquito	40 (21.7	144 (78.3)	184	8.14	5.29	12.52	0.00^***^
8. Putting the water container upside down until the larvae can be seen considering that the lifespan of a mosquito is 1–5 years	27 (52.9)	24 (47.1)	51	0.90	0.50	1.62	0.74^*ns*^
9. Red lime in the container is used if the water container capacity is 100 L	12 (27.9)	31 (72.1)	43	2.91	1.45	5.83	0.00^***^
10. The larval indices surveillance system must be documented on the 25th day of every month in the “Violet book”	39 (21.1)	146 (78.9)	185	8.70	5.63	13.44	0.00^***^

*P* *p*-value of Chi-square test used unless otherwise noted. ^(a)^Fisher’s exact test was used. ^***^p < 0.001 ^**^p < 0.01 ^*^p < 0.05 *ns* Non-significant, *OR* Odd ratio; *CI* Confidence interval

### Comparison of the level of UDS and ULISS among students

Student's good UDS increased from none to 6.8% showing significant change in students’ comprehension towards dengue prevention strategies. Similarly, students with poor understanding of dengue solutions decreased from 50.5% to 42.5%. This indicates the intervention was highly effective in bringing students’ knowledge of UDS.

In terms of ULISS items, students’ good understanding increased from 0.2% to 11.3% and poor understanding decreased from 50.8% to 38%. This shows the effectiveness of education in improving students’ larval indices surveillance system and practices in the manner that students able to manage the population at risk in their village, homes and the school as well (Table 4).

**Table 4:** Comparison of the level of UDS and ULISS among general students

Level	N (%)			OR	95% CI		P
	**Before**	**After**	Total		**Lower**	**Upper**	
UDS Level	239 (50.7)	232 (49.3)	471 (100)	38.08	5.15	281.15	0.000^***^
Good (≥ 80%)	1 (0.2)	32 (6.8)	33 (7.0)				
Poor (< 80%)	238 (50.5)	200 (42.5)	438 (93.0)				
ULSS Level	239 (50.7)	232 (49.3)	471 (100)	70.46	9.65	514.41	0.000^***^
Good (≥ 80%)	1 (0.2)	53 (11.3)	54 (11.5)				
Poor (< 80%)	238 (50.8)	179 (38.0)	417 (88.5)				

^***^p < 0.001 ^**^p < 0.01 ^*^p < 0.05 *ns* Non significant; OR of total: Odd ratio; *95% CI* 95% Confidence interval

### Factors associated with good understanding of dengue solution among students

In the current study, sex and students’ grade was found to be associated with students UDS. It showed female students were over six times (OR = 6.54 (1.54, 27.73), p = 0.002) higher probability of understanding dengue solution than males. Similarly, grade 11 students had a high probability (OR = 12.1 (2.9, 64.9), p = 0.002) to have UDS when compared to grade 10 students (Table 5).

**Table 5:** Factors associated with a good understanding of dengue solutions among high school students

Variables	*Good UDS level*						
		***n (%)***		OR	***95% CI***		***P***
		Before	After		Lower	Upper	
Sex	Male	0 (0.0)	2 (1.5)	1			0.002**
	Female	1 (0.3)	30 (8.8)	6.54	1.54	27.73	
Student grade	10	1 (0.5)	29 (13.7)	1			
	11	0 (0.0)	2 (1.6)	0.098	0.02	0.41	0.002**
	12	0 (0.0)	1 (0.7)	0.045	0.006	0.33	0.002**
Students' experience of UDS at school	Weekly	0 (0)	10 (11.6)	1.79	0.67	4.70	0.24* ns*
	Monthly	1 (0.4)	14 (5.5)	0.85	0.35	2.08	0.73* ns*
	Other	0 (0.0)	8 (6.8)	1			
Students' experience of UDS at home	Weekly	1 (0.9)	7 (6.2)	2.89	0.35	23.92	0.32* ns*
	Monthly	0 (0.0)	24 (8.1)	3.35	0.44	25.50	0.24* ns*
	Others	0 (0.0)	1 (2.6)	1			
Training given	Yes	0 (0.0)	0 (0.0)	0.002	0.000	0.005	0.99* ns*
	No	1 (0.2)	32 (7.0)	1			
Experience of dengue illness in the past 12 months	By themselves	0 (0.0)	1 (3.2)	1.85	0.20	16.50	0.58* ns*
	By neighbors	0 (0.0)	5 (5.8)	1.57	0.09	26.77	0.75* ns*
	By family members	0 (0.0)	1 (5.0)	2.17	0.27	17.05	0.46* ns*
	By others	0 (0.0)	15 (6.8)	1			

*p* *p*-value of Chi-square test used unless otherwise noted. (a)Fisher’s exact test was used. ***p < 0.001 **p < 0.01 *p < 0.05 *ns* Non significant, *OR* Odd ratio; *CI* Confidence interval

### Factors associated with good understanding larval indices surveillance system among students

Female students showed better level of ULISS than males (36.3% vs 13.0%) in the current study but not found to be statistically significant. On the other hand, students’ grades were found to be an important factor associated with good ULISS among students. Grade 11 students were over two times (OR = 2.57 (1.23, 5.37) p: 0.001) more likely to understand larval indices survey when compared to grade 10 students in the current study (Table 6).

**Table 6:** Relationship between individual factors and larval indices surveillance system among high school students

Variables	Good ULSS Level						
		***N (%)***			***95%CI***		***P***
		Before	After	OR	Lower	Upper	
Sex	Male	0 (0.0)	9 (6.8)	1			0.05*
	Female	1 (0.3)	53 (11.5)	2.09	0.99	4.41	
Student grade	10	1 (0.5)	35 (16.6)	2.57	1.23	5.37	0.01**
	11	0 (0.0)	8 (6.4)	0.85	0.32	2.24	0.74* ns*
	12	0 (0.0)	10 (7.4)	1			
Students experience of using ULISS at School	Daily	1 (0.5)	2 (13.3)	1			
	Weekly	0 (0.0)	14 (16.3)	1.26	0.25	6.23	0.77* ns*
	Monthly	1 (0.4)	28 (11.1)	0.84	0.18	3.91	0.82* ns*
	Other	0 (0.0)	9 (7.7)	0.54	0.10	2.78	0.46* ns*
Students experience of using ULISS at home	Daily	0 (0.0)	3 (13.0)	5.70	0.55	58.41	0.14* ns*
	Weekly	1 (0.9)	12 (10.6)	4.94	0.62	39.06	0.13* ns*
	Monthly	0 (0.0)	37 (12.5)	5.42	0.72	40.72	0.10* ns*
	Others	0 (0.0)	1 (2.6)	1			
Training	Yes	0 (0.0)	0 (0.0)	0.01	0.001	0.02	0.00***
	No	1 (0.2)	53 (11.5)	1			
Experience of dengue illness in the past 12 months	By themselves	0 (0.0)	4 (12.9)	1			
	By neighbors	0 (0.0)	8 (9.3)	0.69	0.19	2.48	0.57* ns*
	By family members	0 (0.0)	3 (15.0)	1.19	0.23	5.99	0.83* ns*
	By others	0 (0.0)	25 (11.3)	3.85	0.27	2.65	0.78* ns*

*p p*-value of Chi-square test used unless otherwise noted. (a)Fisher’s exact test was used. ***p < 0.001 **p < 0.01 *p < 0.05 *ns* Non significant, *OR* Odd ratio, *CI* Confidence interval

## Students use of Container Index (CI) at school

**Fig. 2:**
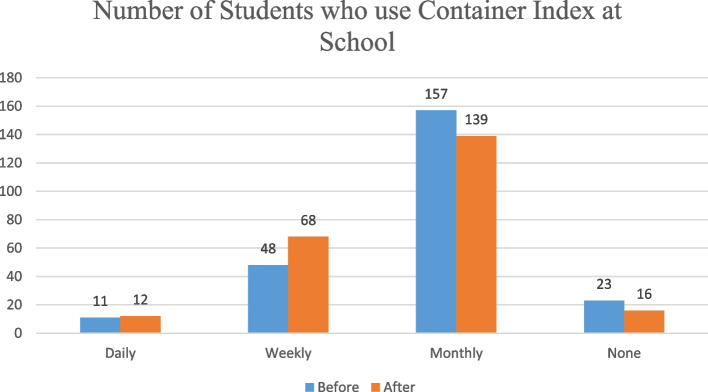
A figure showing the number of students who use container index at high school- on post-COVID-19 in Southern Thailand

This study showed the number of students who used container index in the school has increased from before to after intervention across daily and weekly usage. On the contrary, students who use monthly decreased from before to after the interventions indicating shifting of students toward weekly participation. The school devised three zones for the larval indices survey. The CI was 40% before, and 20% after using the model (Fig. 2).

## Qualitative findings

The thematic analysis of this study showed three important themes of the student’s response. These are learning and information sharing, self-prevention and practical applications, and awareness creation to the community. It also indicated that students in the intervention developed significant learning outcome that made them to be equipped with basic skills to protect themselves and the communities at large. Likewise, the training not only motivated students, but it also created a sese of community responsibility with a high commitment to teaching and sharing information in their circle to enhance overall community wellbeing (Table 7).

**Table 7:** Thematic analysis of the students’ responses

**Themes**	**Meaning**	**Response**
Learning and information sharing	This refers that student learned a lot about dengue prevention (such as identifying signs and symptoms, breeding sites, and how to calculate CI), and information sharing.	*"What I learned from this is how to eliminate mosquito larvae and how to share information with other students in my circle" [Student (S1)].* *“This helped me to identify where dengue usually chose to breed” [Student (S2)].* *“I got confidence on how to prevent dengue and can teach my family and my community at large” [Student (S13)].* *“I shared information about how to manage mosquitoes in our home, neighbors, and entire families” [Student (S14)].* *“I got a good lesson regarding the easy way of preventing mosquito and other vectors from this training in my families and around me” [Student (S5)].* *“I found this training very supportive to enable me to ask and answer the question freely” [Student (S8)].* *“I got many information about dengue, its transmission methods, prevention methods ad whom and how to contact” [Student (S3)].* *“The experience we got from this training gave us confidence to protect ourselves and teach our neighbors on dengue and ways of eliminating mosquito larvae and their prevention methods” [FGD 1].*
Self-Prevention and Practical application	This indicates students’ practical application of protecting their self from the dengue and how to cope the situation through easy self-care remedies and strategies	*“This information gained from the training helped me to apply for our practical day-to-day lives” [Student (S2)].* *"It can be applied at home to keep our loved ones safe from dengue and can also be used to generate income" [Student (S3)]* *“I learned many things about self-protection from dengue and ways of eliminating mosquito and its larvae to best apply this in the family” [Student (S20)].* *“I started destroying mosquito breeding areas in my homes with my families” [Student (S12)].* *“I destroyed mosquito egg-laying site in my homes with family” [Student (S6)].* *“It is possible to change everyone surrounding to prevent mosquito breeding can be applied with no cost to every individuals ” [FGD 2].*
Awareness creation to the community through Education	This implies students’ reflection on the recommendation of raising awareness for the community on dengue prevention methods.	*"What I learned from this is way of educating communities about the causes, risk factors, and prevention methods" [Student (S2)].* *“Educating the communities about dengue is beyond fulfilling for us to protect the public” [Student (S9)].* *“Understanding and teaching the community that even one dengue can able to harm a individuals” [Student (S14)].*

## Discussion

The current study was aimed to evaluate the development and implementation of high school-based dengue solution model in Southern Thailand using CPAR study design.

We utilized four basic steps to develop the model such as preparing, planning, implementing and evaluating by following the guidelines published in earlier studies [23]. Our study findings suggest that students' correct understanding of UDS and ULISS items was well improved after the intervention. This indicates that the provision of UDS and ULISS training for students in the school has brought good knowledge, awareness and behavioral change. This statement goes with findings from rural primary school students of Colombia where students understanding level of UDS and ULISS was found to increase after using the model and resulted in knowledge improvement for both students and their families [24]. Another justification for this could be an increase in dengue information which could have resulted in good dengue knowledge that enables students to understand dengue and its larval indices at schools which is similar to the earliest study from southern Thailand [25]. Therefore, this underscores the need for information and educational communication intervention to scale up the existing efforts and to better decrease dengue incidence.

Developing an effective model such as school based dengue prevention requires using formative research with both program learning and evaluation components through involving all the potential stakeholders in the field such as students, community leaders, public health leaders, researchers, and policy makers. To have good program sustainability, knowledge gained through intervention needs to be transferable to those who need it [26]. Additionally, supporting students in school to have a good understanding of public health solutions will strengthen their existing level of knowledge and best improve the existing public health practices that are in place to respond to rising public health emergencies. This shares explanation of the earlier studies done in southern Thailand [14, 27]. We started developing the model by forming a string (stakeholder) committee, setting up lead students club, school environment assessment, and giving students for UDS and ULISS training. School-based model interventions are the most important and cost effective approach to reach the intended information as required to the target population and to respond the needs of diverse groups such as students, health educators, nurses, teachers and other school community [28, 29]. Additionally, implementing effective public health intervention in the school leads to higher academic achievement, and overall positive school climate [25, 29]. So far, several schools have been engaged in vector control and prevention measures including dengue in various countries and resulted in increased knowledge, improved prevention practices and well contributed to reduced community-based vector transmission [24, 30].

This study showed sex was found to be associated with students UDS. It showed female students were over six times (OR = 6.54 (1.54, 27.73), p = 0.002) higher probability of understanding dengue solution than males. Despite this needs further exploration, it might be due to the higher number of included studies and were more likely to engage in health issues compared with male due to cultural issues and perceived role of taking family responsibility This is in line with findings from Singapore and in Azad Kashmir [31, 32]. Similarly, students’ grades were found to be an important factor associated with good UDS and ULISS among students. Similarly, grade 11 students had a high probability (OR = 12.1 (2.9, 64.9), p = 0.002) to have UDS when compared to grade 10 students. Likewise, Grade 11 students were over two times (OR = 2.57 (1.23, 5.37) more likely to understand larval indices survey when compared to grade 10 students in the current study. This could be due to when students’ educational level increases, their comprehensive understanding about issues such as preventive health also increases. The higher their grade level, the better understanding and engagement of students toward their health and other extracurricular activities. This underscores the importance of tailoring health subjects in the student’s curriculum.

Using CI at the school after the intervention was found to be an important experience that must be shared from this study. This will give good understanding of how stagnant water in the container can contribute to dengue breeding and can be taken as an effective local solution for reducing dengue. Besides, despite the recommendation of CI to be done on weekly basis, significant number of students conducted on monthly basis. This indicated the presence of a gap in adherence among students and underscores the need for educational intervention to improve their adherence.

As a summary, the current study tried to develop and evaluate school-based dengue solution models through mixed method study design using CPAR. However, it has many limitations. Firstly, since the study was only conducted at school setting, it did not capture and cover all effective dengue prevention. Secondly, this study did not assess students level satisfaction after using the model. Thirdly, students who participated in the intervention before and after were not equal due to loss to follow-up (LTF) in the course of the study. Despite this, we tried to show what works locally using community participatory approach. Therefore, we suggest future studies should consider these limitations to improve the robustness of studies on similar topics.

## Conclusion

This participatory action research not only improved students' understanding of dengue but also empowered them to be proactive in various community health initiatives. The positive correlation between educational attainment and understanding of dengue and larval indices survillance underscores the need for tailored educational interventions that address diverse learning needs within the community. Collaborative efforts to establish dengue health information center based at primary schools and above can better improve reduction of dengue incidence.

## Supplementary Information

Supplementary Material 1.

## Data Availability

Data is provided within the manuscript or supplementary information files”

## Abbreviations

BI: Breteau Index
CHV: Community Health Volunteers
CI: Container Index
CPAR: Community Participatory Action Research
EC for DACH: Excellence Center for Dengue and Community Health
FGD: Focus Group Discussion
HI: House Index
KSAO: Kaewsan Sub-district Administration Organization
LI: Larval indices
OR: Odds Ratio
SAO: Sub-district Administration Organization
UDS: Understanding Dengue Solution
ULISS: Understanding larval Indices Surveillance System
VHV: Village Health Volunteers
WHO: World Health Organization
