# Supplementary material for: Evaluation of high school-based dengue solution model in Southern Thailand: a community participatory action research
Source: BMC Public Health. 2024 Nov 28;24:3315. doi: 10.1186/s12889-024-20767-4 (PMC11603923; doi:10.1186/s12889-024-20767-4)
Supplement: Supplementary file 1 — Supplementary Material 1. [file 12889_2024_20767_MOESM1_ESM.docx]

**Appendix I: Student Interview**

S1 What I learned from this activity is how to eliminate mosquito larvae and exchange ideas on methods from younger students, middle school students, high school seniors, and elementary school students. I learned about caring for cattle, caring for patients, and keeping the environment free from diseases like dengue fever.

S2 What I learned from the project is how to apply knowledge to eliminate mosquito larvae in schools, homes, and communities, and to inform people in the community about the causes, problems, and prevention methods.

S3 It can be applied at home to keep our loved ones safe from dengue fever and can also be used to generate income. It helps to eliminate mosquitoes, sustain life, and build basic knowledge of preventing dengue fever and providing basic care when dengue fever occurs. It's worth spending time exchanging diverse knowledge.

S4 I learned about mosquito management, self-protection from mosquitoes, sources of mosquito breeding, and symptoms of dengue fever.

S5 I learned disease prevention from mosquitoes, how mosquito bites cause diseases, and have advised those around me.

S6 I learned mosquito prevention and self-protection and became aware of the dangers posed by mosquitoes.

S7 I learned about the dangers of dengue fever and how to cope, which benefits myself.

S8 I gained knowledge in various aspects including mosquito eradication, preventing mosquito breeding, safe self-care from mosquitoes, knowing breeding sites and sources of mosquito larvae. Importantly, mosquito breeding won't occur if people help by covering containers.

S9 I learned about mosquitoes, how to eliminate them, and what to do when infected with dengue fever, as well as understanding mosquito species.

S10 Methods to prevent mosquito bites.

S11 I learned about mosquito prevention, self-protection, and educating others in the family, understanding the dangers posed by mosquitoes.

S12 I learned about the causes of dengue fever, how to handle patients, eat when sick with dengue fever, the incubation period of mosquito larvae, and how to eliminate them.

S13 I learned about mosquitoes, how to prevent dengue fever, and how to eliminate mosquito larvae, and can advise people in the community and family.

S14 Learning about the history of dengue fever and realizing which gender of mosquitoes cause it, symptoms, fever stages, shock, medical treatment, and importantly, instilling habits to check containers for mosquito eggs, identifying stagnant water areas.

S15 Destroying mosquito breeding grounds, eliminating mosquitoes from water sources.

S16 Mosquito prevention, eliminating mosquito larvae.

S17 Methods of preventing and eliminating mosquitoes, preventing dengue fever, and eliminating mosquito larvae.

S18 Knowledge about project surveys, but most importantly, taking responsibility for assigned tasks, teamwork, unity, and increased courage.

S19 Learned about the causes of dengue fever, what virus types cause it, how many types there are, symptoms, and prevention methods to avoid contracting dengue fever, and learned how to eliminate mosquitoes.

S20 Learned about self-protection from dengue fever and eliminating mosquito larvae, can apply it in the family, and can use the knowledge about dengue fever effectively in daily life.

S21 Has knowledge about mosquito larvae and preventing their occurrence. Preventing the occurrence of mosquito larvae.
